# Beyond Frequency Bands: Complementary-Ensemble-Empirical-Mode-Decomposition-Enhanced Microstate Sequence Non-Randomness Analysis for Aiding Diagnosis and Cognitive Prediction of Dementia

**DOI:** 10.3390/brainsci14050487

**Published:** 2024-05-11

**Authors:** Wang Wan, Zhongze Gu, Chung-Kang Peng, Xingran Cui

**Affiliations:** 1 State Key Laboratory of Digital Medical Engineering, School of Biological Science & Medical Engineering, Southeast University, Nanjing 210096, China; wanwang@seu.edu.cn (W.W.); gu@seu.edu.cn (Z.G.); 2 Center for Nonlinear Dynamics in Medicine, Southeast University, Nanjing 210096, China; ckpeng@dbiom.org; 3 Key Laboratory of Child Development and Learning Science, Ministry of Education, School of Biological Science & Medical Engineering, Southeast University, Nanjing 210096, China

**Keywords:** electroencephalogram, microstate transitions, information-based similarity, non-randomness, dementia

## Abstract

Exploring the spatiotemporal dynamic patterns of multi-channel electroencephalography (EEG) is crucial for interpreting dementia and related cognitive decline. Spatiotemporal patterns of EEG can be described through microstate analysis, which provides a discrete approximation of the continuous electric field patterns generated by the brain cortex. Here, we propose a novel microstate spatiotemporal dynamic indicator, termed the microstate sequence non-randomness index (MSNRI). The essence of the method lies in initially generating a sequence of microstate transition patterns through state space compression of EEG data using microstate analysis. Following this, we assess the non-randomness of these microstate patterns using information-based similarity analysis. The results suggest that this MSNRI metric is a potential marker for distinguishing between health control (HC) and frontotemporal dementia (FTD) (HC vs. FTD: 6.958 vs. 5.756, *p* < 0.01), as well as between HC and populations with Alzheimer’s disease (AD) (HC vs. AD: 6.958 vs. 5.462, *p* < 0.001). Healthy individuals exhibit more complex macroscopic structures and non-random spatiotemporal patterns of microstates, whereas dementia disorders lead to more random spatiotemporal patterns. Additionally, we extend the proposed method by integrating the Complementary Ensemble Empirical Mode Decomposition (CEEMD) method to explore spatiotemporal dynamic patterns of microstates at specific frequency scales. Moreover, we assessed the effectiveness of this innovative method in predicting cognitive scores. The results demonstrate that the incorporation of CEEMD-enhanced microstate dynamic indicators significantly improved the prediction accuracy of Mini-Mental State Examination (MMSE) scores (R^2^ = 0.940). The CEEMD-enhanced MSNRI method not only aids in the exploration of large-scale neural changes in populations with dementia but also offers a robust tool for characterizing the dynamics of EEG microstate transitions and their impact on cognitive function.

## 1. Introduction

Global population aging is rapidly intensifying, leading to a growing number of individuals experiencing cognitive decline. According to estimates by the Global Burden of Disease study, there are currently 57 million people worldwide living with dementia, and it is projected to increase to 153 million in 2050 [1]. Alzheimer’s disease (AD) and frontotemporal dementia (FTD) are progressive neurodegenerative conditions that primarily afflict elderly individuals. AD, the most prevalent form of dementia, constitutes 60–80% of cases, whereas FTD, though less common, accounts for 5–10% of cases [2,3]. Cognitive decline and behavioral changes represent characteristic features of both AD and FTD, albeit affecting the brain in distinct manners.

For an extended period, researchers have shared a common goal of exploring biomarkers or other specific indicators for dementia screening and diagnosis, aiming to replace diagnostic methods based solely on scales or tests. Methods such as positron emission tomography (PET) and magnetic resonance imaging (MRI) can identify certain pathological changes associated with diseases, but they are expensive and have low acceptance rates among patients with non-severe conditions. There is an urgent need for faster and more cost-effective biomarker alternatives to aid in the early detection of dementia.

As technology has advanced, electroencephalography (EEG) has emerged as a crucial tool for investigating and comprehending brain function. Previous studies have highlighted three typical effects on resting-state EEG signals in patients with dementia compared to healthy individuals: diffuse slowing, reduced complexity, and decreased synchrony [4,5]. Changes in the power spectrum, shifting from high-frequency components (alpha, beta, and gamma bands) to low-frequency components (delta and theta bands), are frequently observed in patients with dementia [6]. Reduced complexity refers to a decline in the complexity of brain electrical activity observed in patients with dementia compared to healthy individuals [7]. Additionally, decreased connectivity between cortical areas is seen in many patients with dementia [8].

EEG microstates consider the spatiotemporal evolution of cortical EEG signals, describing the dynamic characteristics of the entire brain neural networks. Renowned for its remarkable resolution in both space and time, microstate analysis finds extensive utility across various domains including dementia [9,10], depression [11], cognitive psychology [12], and sleep [13], facilitating deeper understanding and exploration within these fields. Previous studies predominantly focused on temporal statistical characteristics of microstates, such as duration, occurrence rate, coverage rate, and transition probabilities, while overlooking the broader patterns and characteristics of large-scale changes in brain activity. Recent research efforts have shifted towards investigating dynamic information related to microstate transition patterns. These include analyzing scale-free properties of microstate time series [14], conducting Lempel–Ziv complexity analysis on microstate transitions [15], and exploring microstate entropy rate analysis [16], among others. From the perspective of the brain’s complex systems, there is an urgent need to further investigate analytical methods that can effectively encapsulate the spatio-temporal dynamics of microstates, thereby revealing the dynamic transition patterns inherent in microstate sequences. We intend to employ an information-based similarity analysis tool [17] to detect and quantify certain fundamental patterns embedded in EEG microstates.

Additionally, exploring the spatiotemporal characteristics of brain electrical signals at different frequency scales is also meaningful. Traditional microstate analysis only analyzes the spatio-temporal dynamics of microstates in a wide range of frequency bands and lacks the mining of the spatio-temporal dynamics of EEG microstates in different frequency bands. Researchers have attempted to explicitly decompose the spatiotemporal dynamics of microstates within four discrete, narrowband frequency bands (delta, theta, alpha, and beta) and compare them with classical analyses of broadband signals [18]. Victor Férat et al. argued that microstate analysis with specific frequency decomposition can provide finer-grained spectral information not observable with standard broadband analysis. Although their study was validated in healthy subjects, it is anticipated that this analytical approach will contribute to biomarker research in populations with dementia. Further exploration is required to investigate independent spatiotemporal dynamics indicators across various EEG frequency domain scales, which can be applied to biomarker research in patients with dementia and predictive studies of cognitive function. We intend to investigate the spatio-temporal patterns of EEG microstates across various frequency band scales based on adaptive frequency domain decomposition.

While some prior studies have compared EEG biomarkers between patients with dementia or mild cognitive impairment and healthy controls [15,19,20,21], this study, in addition to investigating EEG microstate dynamic markers in studying health and disease, also focuses on observing the predictive capacity of EEG markers for Mini-Mental State Examination (MMSE) scores.

In light of these challenges, we propose three hypotheses: (1) dementia causes a disruption in the normal transition patterns of EEG microstates, (2) the non-randomness quantification of EEG microstates’ transition patterns can be used to predict cognitive decline scores, and (3) cross-frequency bands microstate analysis can offer more comprehensive insights into dementia disease. To explore these concepts, we introduce a novel method for microstate sequence similarity analysis based on information theory, which captures the microstate sequence non-randomness index (MSNRI) within the underlying patterns of microstates transitions. The effectiveness of this novel approach is evaluated using a publicly available EEG datasets including health controls (HC), FTD, and AD participants [2]. Furthermore, we explored microstate dynamics information across multiple frequencies. Based on the complementary ensemble empirical mode decomposition (CEEMD) [22] method, we introduce a CEEMD-enhanced MSNRI method to provide finer-grained pattern variation information that conventional broadband analysis cannot capture. This research contributes to a more comprehensive understanding of how the dementia brain works, representing a crucial step towards understanding and characterizing the complexity of brain systems.

## 2. Materials and Methods

### 2.1. Dataset

**Figure 1:**
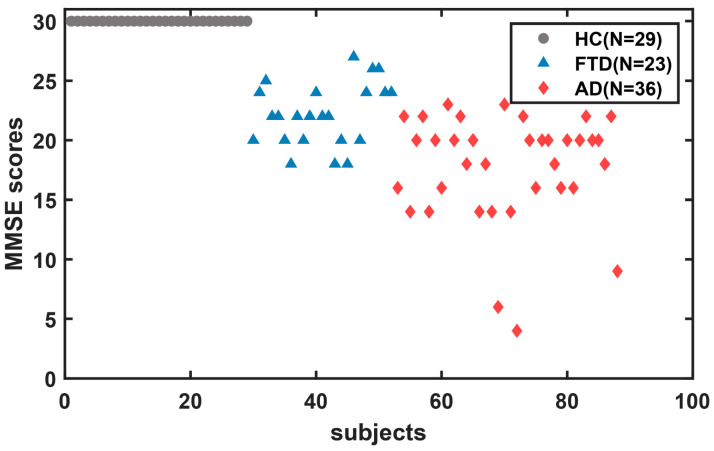
Scatter plot of total MMSE scores for all subjects.

The dataset contains closed-eye resting-state EEG recordings of 88 participants obtained from the Second Neurology Department of the AHEPA General University Hospital in Thessaloniki [2]. Detailed protocol and inclusion criteria are reported in the literature [2]. Thirty-six of them (13 males) were diagnosed with AD, 23 (14 males) were diagnosed with FTD, and 29 (11 males) were healthy controls (HC). The initial diagnosis of patients with AD and FTD was made in accordance with the Diagnostic and Statistical Manual of Mental Disorders, Third Revised Edition (DSM-IIIR, DSM IV, ICD-10) [23], and the criteria provided by the National Institute of Neurological, Communication Disorders and Stroke—Alzheimer’s Disease and Related Disorders Association (NINCDS-ADRDA) [24]. The demographic characteristics are evaluated in Table 1. Cognitive and neuropsychological status was evaluated using the MMSE; the MMSE includes multiple cognitive subfields, such as orientation, memory, attention and calculation, recall ability, and language ability. Total MMSE scores range from 0 to 30, with higher scores indicating better cognitive functioning. MMSE scores of 27–30 indicate normal function, 21–26 indicate mild impairment, 10–20 suggest moderate, and 0–9 suggest severe cognitive impairment. It is important to note that MMSE scores were not the sole criterion for diagnosing cognitive disorders in this study. The scatter plot of total MMSE scores for all subjects is shown in Figure 1, with a mean total MMSE of 17.75 (SD = 4.5) in the AD group, 22.17 (SD = 8.22) in the FTD group, and 30 in the HC group. And the detailed MMSE scores for each subject are shown in Table S1. This study was approved by the Scientific and Ethical Committee of the AHEPA University Hospital of the Aristotle University of Thessaloniki under the protocol number 142/12-04-2023.

The EEG recordings encompass signals from 19 scalp electrodes (Fp1, Fp2, F7, F3, Fz, F4, F8, T3, C3, Cz, C4, T4, T5, P3, Pz, P4, T6, O1, and O2) and 2 reference electrodes (A1, A2), arranged in accordance with the 10–20 international system. Each recording session adhered to a standardized clinical protocol, with participants seated and their eyes closed. Prior to the onset of recording, impedance levels for each electrode were meticulously maintained below 5 kΩ. The recordings were conducted at a sampling rate of 500 Hz, with a resolution of 10 uV/mm. For the AD group, recording sessions lasted approximately 13.5 min each, ranging from 5.1 to 21.3 min. In the FTD group, sessions lasted around 12 min per session, ranging from 7.9 to 16.9 min. For the HC group, recording sessions lasted approximately 13.8 min each, with durations ranging from 12.5 to 16.5 min. Overall, the dataset comprised a cumulative duration of 485.5 min for AD recordings, 276.5 min for FTD recordings, and 402 min for HC recordings.

### 2.2. Preprocessing

The preprocessing of EEG signals was conducted using the Fieldtrip toolbox (version 20220215) implemented in Matlab R2023a [25]. The EEG signals were filtered using a notch filter with a frequency range of 49.8–50.2 Hz, along with a 5th order high-pass filter with a cutoff frequency of 0.3 Hz and a 5th order low-pass filter with a cutoff frequency of 70 Hz.

The EEG data were subsequently down-sampled to 250 Hz and re-referenced to the average reference. After filtering, we conducted a visual inspection of signal quality and removed segments with significant artifacts. The eye movements, eye blinks, or heartbeat-related signal contaminants were removed through infomax independent component analysis (ICA) with the “fastica” method [26]. The preprocessed data for each participant, comprising 180 s of data, were selected for subsequent analysis.

### 2.3. EEG Microstate Analysis

We performed the EEG microstate analysis using the functions provided by Microstate EEGlab Toolbox (version 1.0) [27]. We first extracted the peak of the Global Field Power (GFP), setting the minimum peak distance to 10 ms, and configuring the number of GFP peaks for segmentation for each participant to be 2000. Additionally, any GFP peaks exceeding one standard deviation of the GFPs across all maps were excluded from consideration. For microstate segmentation, we employed a modified K-means algorithm, disregarding the polarity of EEG topographies during clustering [28,29]. Here, we set the number of microstates to four based on previous findings from methods employing K-means clustering, which demonstrated, through cross-validation, that this number yielded optimal clustering with high reproducibility [30,31]. We configured the algorithm with 50 random initializations and a maximum of 1000 iterations. Additionally, the threshold of convergence based on relative change in noise variance was set to 1 × 10^−6^. The original toolkit documentation includes a complete set of sample code for microstate analysis.

Then, the clustered stable-state templates were reverse-fitted onto the EEG or IMFs of each participant, marking the microstate categories for segments of EEG or IMFs. Additionally, to mitigate short-term continuous changes in microstate labeling caused by noise, microstates with durations of less than 30 ms were classified as the next most probable microstate class based on global map dissimilarity (GMD). Next, we computed classical microstate dynamic characteristics, including duration (the average duration of a microstate activity in milliseconds), occurrence (the frequency of occurrence of a particular microstate), and coverage (the percentage of time that each microstate category occupies throughout the entire period of analysis).

When evaluating the microstate templates for health and disease, we created group-level microstate templates separately for the HC group, FTD group, and AD group, and global maps between groups were aligned by calculating correlation coefficients of the maps and visual inspection. However, in subsequent analyses of microstate indicators, we uniformly employed the microstate template of all participants to reverse-fit the data of each individual participant.

### 2.4. CEEMD-Enhanced Microstate Sequence Non-Randomness Analysis

**Figure 2:**
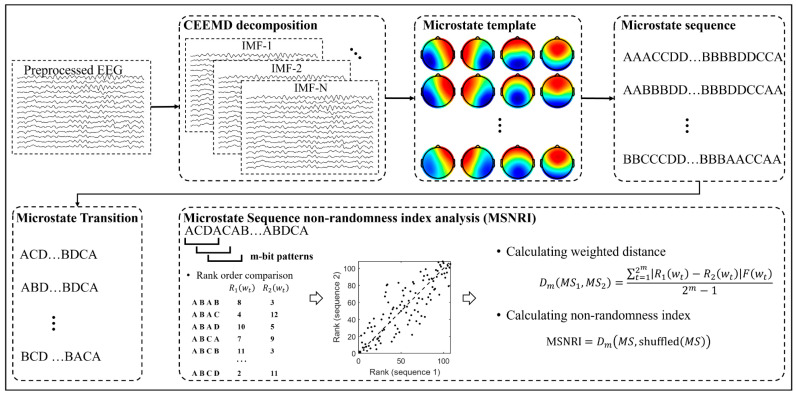
Schematic flow of CEEMD-enhanced microstate sequences non-randomness index analysis. Step 1: decompose broadband EEG signals into a sequence of Intrinsic Mode Functions (IMFs) using CEEMD. Step 2: perform the microstate analysis on different IMFs and reconstruct the microstate sequence based on the microstate topographic template at each IMF (Red and blue indicate positive and negative values, respectively). Step 3: construct microstate transition sequences from microstate sequences. Step 4: calculate the microstate sequence non-randomness index according to the proposed methodology.

Here we propose a novel measure of EEG microstate transitioning, called microstate sequence non-randomness analysis. In addition to studying the spatio-temporal patterns of microstates at a broadband scale or at narrowband frequency bands (delta, theta, alpha, and beta), this study explores the dynamic patterns of microstates at multiple adaptive frequency scales applying CEEMD. The schematic flow of CEEMD-enhanced microstate sequences non-randomness analysis is shown in Figure 2.

Prior to conducting microstate analysis, broadband EEG signals are decomposed into a sequence of Intrinsic Mode Functions (IMFs) using CEEMD. These IMFs elucidate the oscillatory patterns of EEG across various frequency scales, enabling microstate analysis to be conducted at different IMF scales thereafter.

**Figure 3:**
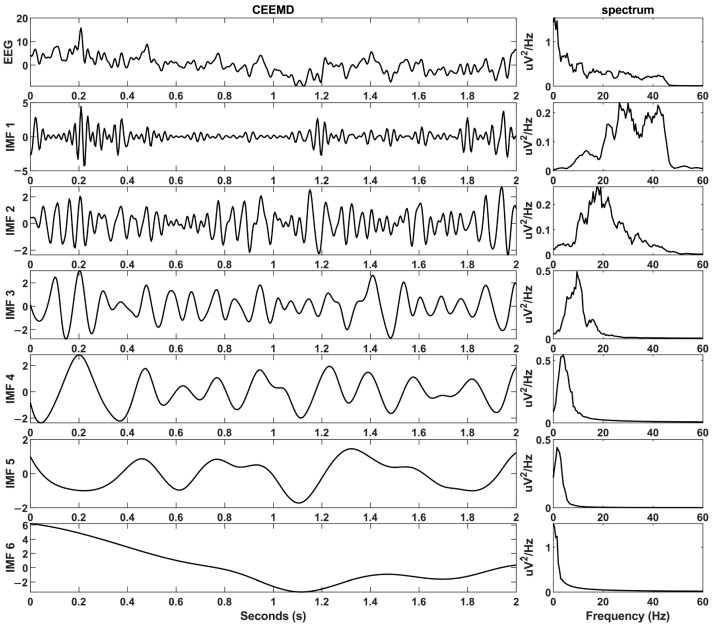
Waveform and corresponding spectrum graphs of IMF components after CEEMD decomposition.

The CEEMD proposed by Yeh et al. [22] effectively addresses the mode mixing issue of the Empirical Mode Decomposition (EMD) algorithm and the noise residue problem of the Ensemble Empirical Mode Decomposition (EEMD) algorithm. CEEMD and EEMD have similar computational processes, with the difference being that EEMD adds white noise only once each time, while the CEEMD algorithm adds white noise to the original signal and subtracts white noise from the original signal. Both signals undergo EMD decomposition simultaneously, and the average is found to counteract the noise added to the signal.

(1) $$\left[\begin{array}{c} M1\\ M2 \end{array}\right]=\left[\begin{array}{c} 1\;\;\;\;\;1\\ 1-1 \end{array}\right]\left[\begin{array}{c} S\\ N \end{array}\right],$$

where, S represents the original signal, N represents the added white noise, M1 is the original data with added white noise, and M2 is the original signal with subtracted white noise. Then, EMD decomposition is performed separately on the signals with added noise and subtracted noise. The resulting two sets of IMFs are averaged to obtain the final IMF component. In this study, the standard deviation of the added white noise is set to 0.1, and the number of iterations is set to 100. The waveform and corresponding spectrum results of the IMF components after CEEMD decomposition are displayed in Figure 3.

EEG IMF frames are labeled based on their best matching group template using a “winner takes all” strategy (frames with correlations below 0.5 are not labeled), thereby generating microstate sequences. We first transformed the microstate sequence into a sequence without duplicate labels, for example, transforming a sequence like AACCDDBBBBDDCCA into ACDBDCA. This transformation overlooks the duration of microstates, rooted in the notion that transitions between different brain states measured through EEG microstates convey fundamental information about brain activity [28].

Following that, we utilized the Information-based Similarity (IBS) analysis, a tool rooted in physics and statistical linguistics [32], to detect and quantify the inherent patterns of microstate dynamic transitions. Subsequently, the microstate transition sequence MS was partitioned into numerous sets of symbolic sequences $w_{t}$ with length *m*, called *m*-bit “word”. Each *m*-bit word, denoted as $w_{t}$, signifies a particular change pattern within cortical EEG IMFs. Through the incremental shifting of a single symbolic point, a sequence of m-bit words is generated, resulting in $2^{m}$ distinct change patterns for each microstate transition sequence. The frequency of occurrence for each word is tabulated, and all *m*-bit words are then arranged in descending order based on their respective frequencies. Subsequently, the change pattern sequences (“words”) present in both microstate symbolic sequences are singled out for scrutinizing the information-based similarity between two EEG signals.

$p_{1}\left(w_{t}\right)$ and $R_{1}\left(w_{t}\right)$ represent the probability and rank of the *m*-bit word $w_{t}$ in the symbolic transition sequence ${MS}_{1}$, and $p_{2}\left(w_{t}\right)$ and $R_{2}\left(w_{t}\right)$ represent the probability and rank of the same word $w_{t}$ in the sequence ${MS}_{2}$, respectively. The rank of each m-bit word in the first symbolic sequence is plotted against its rank in the second symbolic sequence, creating a rank order comparison graph. When two symbolic series exhibit a similar arrangement, the scatter points will cluster around the diagonal line. We defined the microstate sequence IBS distance $D_{MSIBS}\left({MS}_{1},{MS}_{2}\right)$ between ${MS}_{1}$ and ${MS}_{2}$ as follows:

(2) $$D_{MSIBS}\left({MS}_{1},{MS}_{2}\right)=\frac{\sum_{t=1}^{2^{m}}\left|R_{1}\left(w_{t}\right)-R_{2}\left(w_{t}\right)\right|F\left(w_{t}\right)}{2^{m}-1},$$

(3) $$F\left(w_{t}\right)=\frac{1}{Z}\left[-p_{1}\left(w_{t}\right)logp_{1}\left(w_{t}\right)-p_{2}(w_{t})logp_{2}(w_{t})\right],$$

(4) $$Z=\sum_{t}\left[-p_{1}\left(w_{t}\right)logp_{1}\left(w_{t}\right)-p_{2}(w_{t})logp_{2}(w_{t})\right],$$

$F\left(w_{t}\right)$ represent the weight of each *m*-bit word, computed through Shannon’s entropy and normalized with the normalization factor Z. Given that not all words carry equal weight in the rank frequency distribution, a weight function $F\left(w_{t}\right)$ must be factored in when computing IBS distance.

Finally, we proposed a method to estimate the degree of complexity or structural richness of spatiotemporal changes in EEG microstate. To this end, we generated alternative symbolic transition sequences by randomly shuffling the microstate transition sequence Y. Random shuffling of the data produces exactly the same distribution as the original sequence but destroys the sequential ordering. The microstate sequence non-randomness index (MSNRI) was defined as the distance between an EEG spatiotemporal symbolization sequence and its random alternatives,

(5) $$MSNRI{=D}_{MSIBS}\left(MS,shuffled\left(MS\right)\right),$$

The closer the distance between the original microstate dynamic sequence and its disordered sequence, the smaller the MSNRI. On the contrary, the farther the distance, the greater the MSNRI. The different IMFs were analyzed separately to obtain the CEEMD-enhanced MSNRI.

### 2.5. Microstate Sequence Lempel–Ziv Complexity

Calculating the nonlinear complexity of transition sequences using the Lempel–Ziv compression algorithm has been a commonly used method in previous studies [15,16]. The Lempel–Ziv complexity measurement quantifies the number of sub-words that can be found in the entire sequence, assessing the richness of a sequence with a finite number of generating elements. In this study, the calculation of microstate sequence Lempel–Ziv complexity (MSLZC) follows the method proposed by Michael Lassi et al. [9] The MSLZC is computed by measuring the length of the encoded dictionary in the microstate sequence using the *zlib* Python package (version 1.3.1), after excluding the Huffman coding component. To address variations in sequence length, normalization by the total length of the signal is conducted.

### 2.6. Statistical Analysis

To explore the potential disparity between the EEG microstate features of health and patients, as well as to prove the probable significance of our proposed methodology, we employ the Mann–Whitney U test on our datasets. The Mann–Whitney U test is a nonparametric method enabling us to ascertain whether the statistics under consideration, in our case, synchrony results, exhibit differing values across two distinct populations. Significant difference was defined as a *p*-value < 0.05. All statistical analysis methods were performed in MATLAB R2023a.

### 2.7. Predictive Models for the MMSE Score

We developed a predictive model for MMSE total scores based on resting EEG microstate metrics to explore the possibility of resting-state EEG microstate dynamics characterization to replace or supplement MMSE screening. For feature preprocessing, all predictor variables were normalized from 0 to 1. To fully capture the contribution of microstate dynamics features to predict MMSE scores, we used multiple linear regression to estimate MMSE scores.

In order to demonstrate the effectiveness of the predictive model in real-world scenarios and to ensure the stability and accuracy of the prediction outcomes, we employed the Leave-One-Subject-Out (LOSO) cross validation method. For the LOSO classification experiment, all data from all subjects except one was used for training, with the excluded subject’s data reserved for testing. For a dataset containing N samples, LOSO performed N iterations. Specifically, for the MMSE score prediction model for all subjects (N = 88), the LOSO approach required 88 iterations for validation, with 87 subjects as the training set and the remaining 1 subject as the test set for each iteration. Finally, the coefficient of determination (R-squared), mean squared error (MSE), root mean squared error (RMSE), and mean absolute error (MAE) metrics were utilized to assess the consistency between the actual MMSE scores and the predicted MMSE scores by the prediction model.

## 3. Results

### 3.1. Microstate Maps Analysis

**Figure 4:**
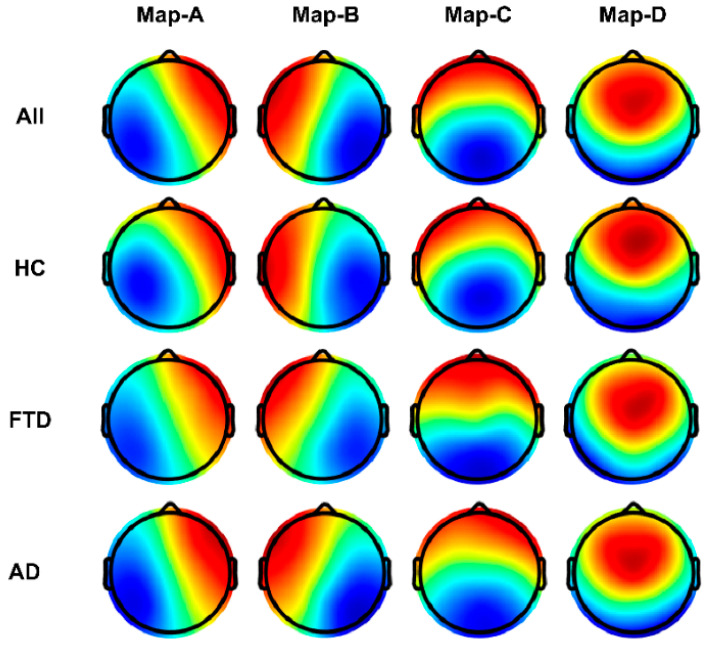
Microstate template topographies for the four classes. (**Top**) Globally clustered maps for all cohorts for maps A–D from left to right. (**Second**) As above, but for the HC group. (**Third**) For the FTD group. (**Bottom**) For the AD group. Red and blue indicate positive and negative values, respectively. The polarity is ignored during microstate analysis.

The topology of each of the four microstate categories in the data separately for all subjects, the healthy group, the FTD group, and the AD group is shown in Figure 4. In the HC group, these microstate classes closely correspond to the classical categories A–D, which are linked to wakeful rest and represent electrophysiological manifestations of the auditory (Map-A), visual (Map-B), salience (Map-C), and frontal working memory/attention (Map-D) resting state networks [28].

**Figure 5:**
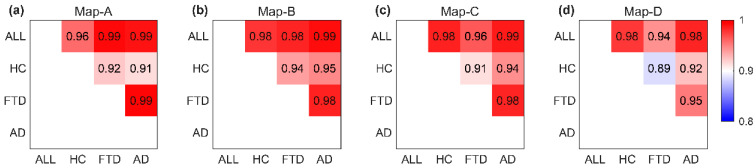
Spatial correlation between microstate template maps of the same class across groups. The allocation of microstate maps assigned to the same grade (**a**–**d**) was compared across all subject groups (All, HC, FTD, AD). Each element of every matrix displays the spatial correlation coefficient between two maps (numeric values within the squares, represented by colors on a color bar).

Spatial correlation between microstate topographies measured by the absolute value of spatial cross-correlation coefficients reveals subtle differences between healthy and diseased groups. Figure 5 illustrates the topological correlation comparison matrix between microstate template maps of the same category across different groups (all groups, HC, FTD, AD). The largest difference was observed for Map-D between HC and disease groups (Table 2, average correlation of 0.921 for Map-D).

Finally, spatial correlation between the microstate templates identified for all people (All) and those identified for each subject was calculated using a segment temporal smoothing constraint of 5 samples (20 ms). EEG frames are labeled based on their best matching group template using a “winner takes all” strategy (frames with correlations below 0.5 are not labeled), thereby generating microstate sequences for each participant, each comprising 45,000 symbols (180 s multiplied by 250 Hz) for further analysis.

### 3.2. EEG Microstate Sequence Analysis

**Figure 6:**
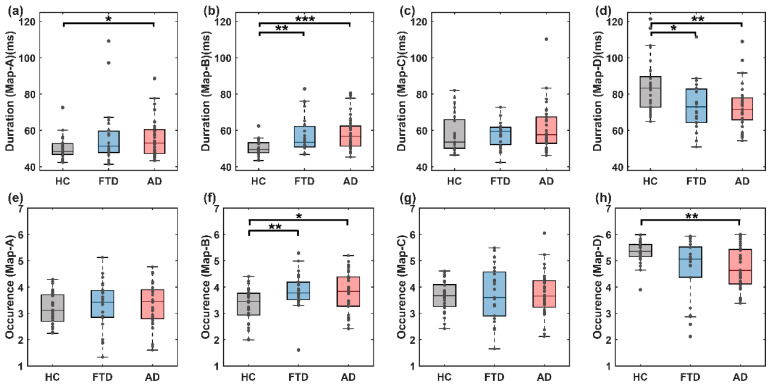
Classical dynamic characteristics of microstates. (**a**–**d**) Duration of microstates in the HC, FTD and AD groups; (**e**–**h**) occurrence of microstates in the HC, FTD and AD groups. In all panels, the significance of the corresponding statistical test is represented as follows: * *p* < 0.05, ** *p* < 0.01, *** *p* < 0.001.

We meticulously examined the temporal dynamics of EEG microstates, specifically focusing on the mean duration (Figure 6a–d) and occurrence (Figure 6e–h) of each microstate among different groups. Our results, detailed in Figure 6, revealed significant disparities in the temporal properties of the microstates when comparing the HC group to both the FTD and AD groups.

For microstate B, the disease groups exhibited a pronounced increase in both the mean duration and occurrence, as illustrated by the following statistics: HC (50.180 ms) versus FTD (57.485 ms) with a non-parametric Mann–Whitney U test (z = −3.058, *p* = 0.002), and HC versus AD with a duration of 50.180 ms versus 58.223 ms (z = −4.058, *p* < 0.001). Similarly, the occurrence parameters were significantly higher in the disease groups, with 3.318 for HC compared to 3.862 for FTD (z = −2.763, *p* = 0.006) and 3.318 for HC compared to 3.777 for AD (z = −2.408, *p* = 0.016).

Conversely, microstate D showed a significant decrease in both metrics within the disease groups compared to the HC group: HC versus FTD with a duration of 84.160 ms versus 75.397 ms (z = 2.506, *p* = 0.012), and HC (84.160 ms) versus AD (75.348 ms) (z = 2989, *p* = 0.003). The occurrence parameters were only significantly reduced in the AD group (HC vs. AD: 5.333 vs. 4.725, z = 3.082, *p* = 0.002). No significant differences in the time-domain characteristics between the FTD and AD groups were found in any of the microstate categories.

**Figure 7:**
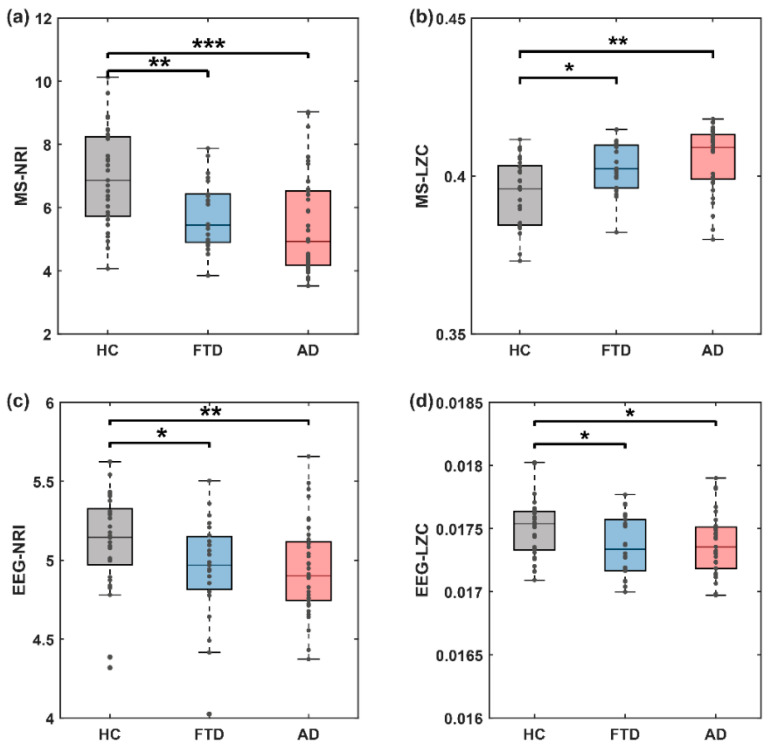
Microstate sequence dynamic statistics are significantly altered in disease condition (both FTD and AD). (**a**) Microstate sequence NRI, (**b**) microstate sequence LZC, (**c**) EEG time series NRI, (**d**) EEG time series LZC. Stars denote effect size of Mann–Whitney U test: * *p* < 0.05, ** *p* < 0.01, *** *p* < 0.001. Points in the boxplots show values for each participant. (MSNRI with parameter m = 8).

We next explored whether the transition behavior of microstate sequences was altered. Researches have shown that the transitions of microstate sequence are non-smooth, non-Markovian, and scale-free [14]. We therefore calculated the MSNRI and MSLZC of the microstate transitioning sequenc. Compared with the HC group, the MSNRI was markedly reduced in both the FTD group (6.958 vs. 5.756, z = 2.805, *p* = 0.005, Figure 7a) and AD group (6.958 vs. 5.462, z = 3.426, *p* = 0.0006, Figure 7a), suggesting the presence of abnormal microstate pattern transitions in the EEGs of patients with cognitive impairment. Furthermore, this effect size was notably larger compared to other used measures of EEG microstate complexity utilized in previous studies on AD or MCI (Mild Cognitive Impairment), such as MSLZC (HC vs. FTD: 0.394 vs. 0.405, z = −2.414, *p* = 0.016, HC vs. AD: 0.394 vs. 0.403, z = −3.200, *p* = 0.001, Figure 7a). Meanwhile, we also compared the results of NRI and LZC computed on the EEG time series (EEG-NRI and EEG-LZC), both of which had weaker intergroup differences than those analyzed on the microstate transition series (Figure 7c,d).

### 3.3. CEEMD-Enhanced Non-Randomness Analysis of Microstate Dynamic Patterns

**Figure 8:**
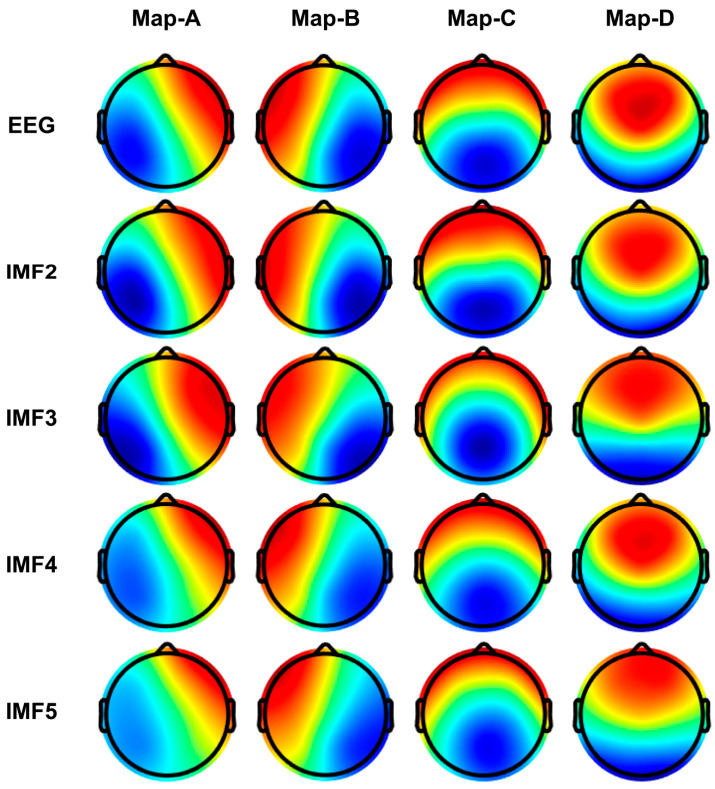
Microstate template topographies for each IMF after CEEMD decomposition. Red and blue indicate positive and negative values, respectively.

**Figure 9:**
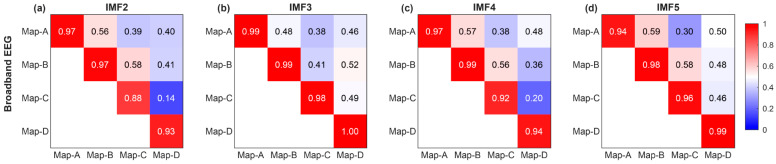
Spatial correlation between microstate topographies across IMFs. Spatial correlation of microstate template topographies (**a**) between the broadband EEG and IMF 2, (**b**) between the broadband EEG and IMF 3, (**c**) between the broadband EEG and IMF 4, (**d**) between the broadband EEG and IMF 5. Numeric values within the squares, represented by colors on a color bar.

Next, we expanded the EEG into various IMFs using CEEMD decomposition, aiming to gain insights into the microstate spatio-temporal dynamics of different IMFs. Figure 8 illustrates the topographic results of microstate segmentations in the different IMF bands (IMF2, IMF3, IMF4, and IMF5). We found that these microstate topologies on different IMFs are very similar to those on the full-band EEG, all conforming to previously specified prototypes in the literature. As shown in Figure 9, all spatial correlations are very high when comparing the topology of the microstate templates between the broadband EEG and each IMFs (i.e., diagonal entries in the correlation matrix), which provides a rationale for reconstructing the sequence of microstate transitions within the different IMFs using their respective microstate templates.

**Figure 10:**
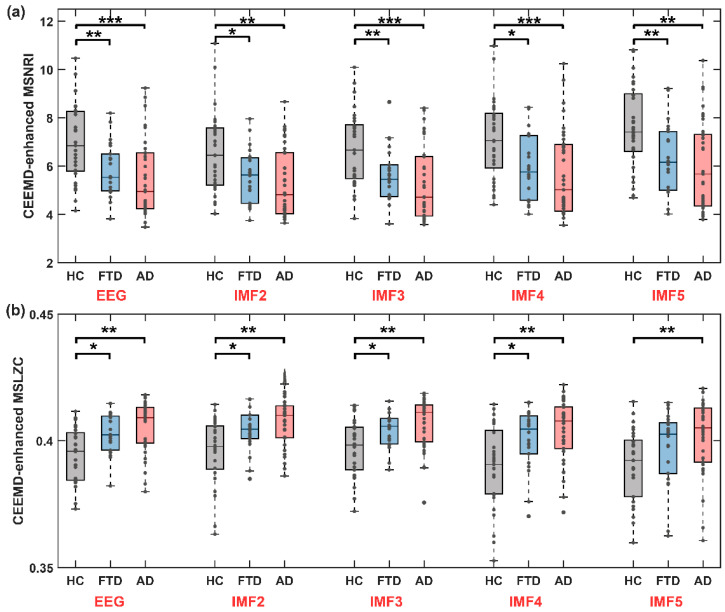
Microstate sequence dynamic statistics are significantly altered in disease condition (both FTD and AD) in all IMFs after CEEMD decomposition. (**a**) The CEEMD-enhanced MSNRI among the HC, FTD, and AD groups (MSNRI with parameter m = 8). (**b**) The CEEMD-enhanced MSLZC among the HC, FTD, and AD groups. Stars denote effect size of Mann–Whitney U test: * *p* < 0.05, ** *p* < 0.01, *** *p* < 0.001.

Here, we obtained the sequence of microstate transitions specific unique to each IMF through a conventional microstate analysis conducted on individual IMF components. Subsequently, we obtained MSNRI and MSLZC for each distinct IMF to discern variations in microstate dynamic patterns among the HC, FTD, and AD groups across different IMF components. The analysis results of the MSNRI and MSLZC indices across IMF components for the HC, FTD, and AD groups are illustrated in Figure 10. The detailed results of MSNRI and MSLZC for the HC, FTD, and AD groups across different IMF scales are shown in Table 3.

Across all IMF components, the HC group consistently exhibited the highest MS-NRI, followed by the FTD group, and the lowest was observed in the AD group. Notably, in all the IMF components included in the analysis, the MS-NRI index for the AD group was significantly lower than that for the HC group (IMF2: z-value = 3.196, *p* = 0.001, IMF3: z-value = 3.507, *p* < 0.001, IMF4: z-value = 3.304, *p* = 0.001, IMF5: z-value = 3.264, *p* = 0.001). For the FTD group, there was a significant decrease in MS-NRI compared to the HC group across the scales of IMF2 to IMF5.

It is noteworthy that the trend in MSLZC contrasted with that of MSNRI. Across all IMF components, the AD group consistently demonstrated the highest MSLZC, followed by the FTD group, while the HC group consistently displayed the lowest. Although MSLZC across different IMF scales also exhibited significant intergroup differences, the MSNRI index proposed in this study statistically demonstrated more significant distinctions in characterizing differences between healthy individuals and those with cognitive impairment disorders. The findings imply that conducting cross-frequency microstate dynamic analysis could provide further valuable insights into diagnosing and predicting cognitive impairment disorders.

### 3.4. Microstate Sequence Non-Randomness Has Predictive Power for MMSE Scores

To better understand how well the CEEMD-enhanced non-randomness analysis method can aiding diagnosis and cognitive prediction of Alzheimer’s disease, we developed a predictive model for MMSE scores based on EEG microstate metrics to explore the possibility of resting-state EEG to replace or supplement MMSE screening. The baseline method incorporates temporal statistical features of microstates extracted from resting-state EEG, along with MSLZC and MSNRI (with parameter m from 4 to 8). By contrast, the proposed method in this paper includes CEEMD-enhanced MSLZC and MSNRI (with parameter m from 4 to 8), along with the integration of microstate temporal statistical indicators.

**Figure 11:**
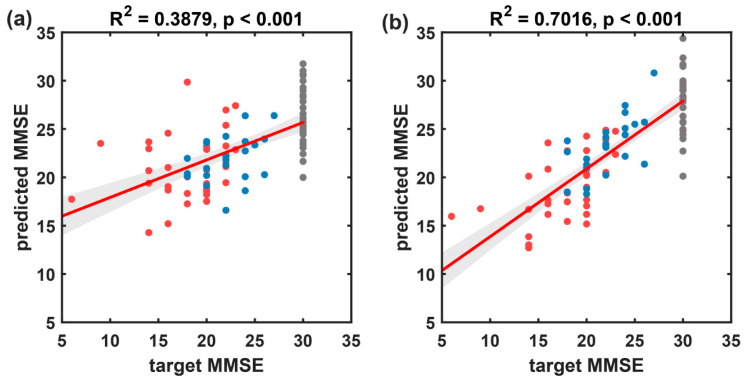
The results of the multivariate linear regression model predicting MMSE scores using resting-state EEG indices for all participants, with target MMSE scores on the *x*-axis and predicted MMSE scores on the *y*-axis. (**a**) Scatter plot and fitted line of the baseline model’s predicted values and true values based on microstate temporal metrics. (**b**) Scatter plot and fitted line of the prediction model’s predicted values and true values based on the microstate sequence dynamic metrics proposed in this paper. Black circles represent the HC group, blue circles represent the FTD group, and red circles represent the AD group. The solid red line represents the scatter fit curve, and the shaded area indicates the 95% confidence interval.

The results of the multivariate linear regression prediction model based on MMSE scores for all subject samples are shown in Figure 11 and Table 4. Compared to the baseline method (R^2^ = 0.388, rMSE of 4.784, and MAE of 3.511), the proposed method in this paper achieved superior predictive performance (R^2^ = 0.702, rMSE of 3.340, and MAE of 2.555).

**Figure 12:**
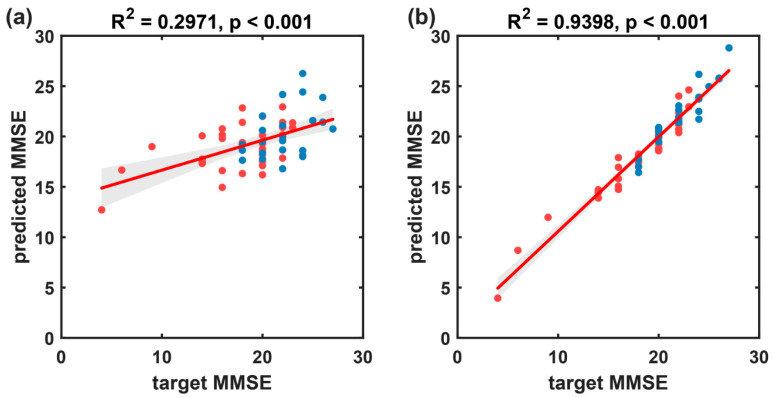
The prediction results of the multivariate linear regression model, which predicts MMSE scores based on resting-state EEG indices, for the cognitive impairment group (FTD and AD). (**a**) Scatter plot and fitted line of the baseline model’s predicted values and true values based on microstate temporal metrics. (**b**) Scatter plot and fitted line of the prediction model’s predicted values and true values based on the microstate sequence dynamic metrics proposed in this paper. Blue circles represent the FTD group, and red circles represent the AD group. The solid red line represents the scatter fit curve, and the shaded area indicates the 95% confidence interval.

From the scatter plot of predicted MMSE scores against target MMSE scores (Figure 11), it is evident that the MMSE scores for the HC group are all 30, forming a cluster on the plot, which might have influenced the overall performance of the predictive model. Therefore, this subsection further establishes a multivariate linear regression prediction model for MMSE scores based on participants with cognitive impairment disorders. The results are illustrated in Figure 12 and summarized in Table 5. On the disease group, the proposed method in this paper exhibited significantly improved predictive performance (R^2^ = 0.940, rMSE of 1.077, and MAE of 0.807) compared to the baseline method (R^2^ = 0.297, rMSE of 3.680, and MAE of 2.877).

The results of the multivariate linear regression prediction model demonstrate that utilizing resting-state EEG microstate indicators can achieve high accuracy in the cross-subject prediction of MMSE scores, confirming the potential of EEG biomarkers as substitutes or complements to MMSE. Additionally, the exploration of non-randomness and complexity indices of microstate spatio-temporal patterns enhanced by CEEMD holds significant importance in assessing the severity of cognitive impairment.

## 4. Discussion

In this study, our core hypothesis is that the disruption in the equilibrium between spatiotemporal pattern transitions in brain microstates constitutes a crucial characteristic of the dementia process. Furthermore, we postulate that the patterns of transitions between EEG microstate may be biomarkers to predict cognitive abilities. To test this hypothesis, we developed a microstate sequence non-randomness index and gauged its efficacy in distinguishing HC and dementia diseases (FTD and AD). Our research findings indicate that this method exhibits highly significant differences in distinguishing between healthy subjects and patients with dementia, providing compelling evidence that spatiotemporal patterns of microstates in resting-state EEG signals can serve as valuable biomarkers for dementia.

This investigation uncovers another significant revelation. Cross-frequency microstate dynamic analysis, CEEMD-enhanced MSNRI, expands the differences between healthy and dementia states, significantly enhancing cognitive function prediction performance, making it a reasonable complement to broadband microstate analysis. These findings carry practical significance, suggesting that EEG can offer an alternative avenue for assessing cognitive diseases or cognitive abilities.

In this work, we present a novel method of non-randomness to microstate transition sequences since microstate transitioning is non-linear, non-stationary, and non-Markovian [14]. As anticipated [9], maintaining the balance and stability of brain spatiotemporal pattern transitions plays a crucial role in human cognitive health. We found significant differences in the non-randomness of spatiotemporal patterns of microstates between healthy individuals and patients with cognitive impairment disorders. The MSNRI of patients with dementia decreases, indicating a shift towards more randomness in the spatiotemporal patterns of their brain’s complex system (Figure 7a, HC vs. FTD vs. AD: 6.958 vs. 5.756 vs. 5.462). Interestingly, MSNRI will decline more in AD than in FTD. In addition, the CEEMD-enhanced MSNRI produced consistent results at every IMF scale (Figure 10a, all IMF scales). In a healthy state, the spatiotemporal patterns of brain microstates exhibit a more complex macroscopic structure and demonstrate the lowest level of randomness. Conversely, dementia diseases lead to increased randomness in brain spatiotemporal patterns. This understanding contributes to a deeper comprehension of the neural mechanisms underlying cognitive impairment disorders and provides crucial clues for related research and clinical diagnosis.

MSLZC is a previously reported measurement for studying microstate transitions [9,15,16]. In this study, we also observed differences in MSLZC between the healthy group and the group with the disease, but the significance of the differences was weaker compared to MSNRI. We observed a significant increase in MSLZC tendency in the AD group (Figure 7 and Figure 10). It is worth noting that, in comparison with another MSLZC-based analysis of AD patients, our results for the LZC analysis of the EEG time series were consistent, but the results for the MSLZC analysis of the microstate series were reversed (MSLZC was reduced in AD, *p* = 0.0023) [15]. Possible reasons for this discrepancy include (1) differences in the length of data included in the analysis (we used 180 s, while they used 20 s), (2) differences in microstate segmentation methods, and (3) variations in the severity of AD; in their study, the average MMSE score for AD was 23, whereas ours was 17.75. The U-shaped relationship between complexity and disease severity might lead to such differences in results [33]. Furthermore, the biological significance of LZC may differ from that indicated by other microstate indicators [34]. For instance, in other studies, MSLZC was found to be higher in patients with depression than in controls [35], and LZC in patients with thalamic ischemic stroke has been found to be higher compared to the controls [36].

Meanwhile, the CEEMD-enhanced MSNRI algorithm is more effective compared to broad frequency domain scale analysis (Figure 10 and Figure 12 and Table 3). In this study, CEEMD was used to refine the frequency domain of EEG signals into multiple scales. Compared to Fourier transform, CEEMD can analyze nonlinear and non-stationary signals. Unlike wavelet transform, CEEMD does not select basis functions. It adaptively extracts the intrinsic mode functions of the signal while simultaneously separating trend components and noise, which helps capture patterns in the specific frequency scales of EEG signals from patients with cognitive impairment disorders.

After the introduction of the CEEMD-based MSNRI and MSLZC indicators, compared to traditional microstate temporal features (Figure 12a, R^2^ = 0.297, rMSE of 3.680, and MAE of 2.877), the method integrating CEEMD-enhanced MSNRI and MSLZC shows a significant improvement in the accuracy of predicting MMSE scores (Figure 12b, R^2^ = 0.940, rMSE of 1.077, and MAE of 0.807). As shown in Table 6, this prediction performance exceeds the results of previous studies of MMSE prediction using microstate or other EEG characterization methods [20,37,38,39,40,41], while also confirming the potential of resting-state EEG biomarkers as substitutes or supplements for MMSE.

This research also has several limitations. One notable limitation concerns the necessary length of the data. Since we were analyzing pattern information within EEG microstate transition sequences, the microstate sequences were reduced to transition sequences during the analysis process. As a result, a relatively longer data length is required to obtain sufficient information. In this article, we used a data length of 180 s. This is in contrast to other methods that may only require several seconds or tens of seconds of EEG data to complete feature extraction. Secondly, we employed a relatively small clinical dataset (29 healthy individuals, 59 patients with dementia) for screening diseases. While this validated the effectiveness of the algorithm to some extent, further validation of its robustness in cognitive disease screening applications requires larger datasets, which will be pursued in future work. Additionally, in predicting cognitive test scores, we only utilized EEG markers to predict MMSE scores. While MMSE exhibits acceptable accuracy for dementia screening, its specificity and sensitivity are lower for milder cognitive impairments such as mild cognitive impairment because some individuals with early cognitive decline can still score within the normal range, meaning that it may not be sensitive enough to detect subtle cognitive changes that occur in the early stages of MCI. Therefore, future work needs to validate the ability of EEG markers to predict scores from other computerized tests assessing early cognitive decline.

## 5. Conclusions

The proposed novel CEEMD-based MSNRI approach employs topographic space compression, physics, and statistical linguistic tools to detect and quantify certain fundamental patterns embedded in EEG microstates beyond frequency bands. By leveraging EEG datasets from HC, FTD, and AD participants, we validated our hypotheses. Our findings revealed that healthy individuals exhibit more complex macroscopic structures and non-random spatiotemporal patterns of microstates, whereas dementia disorders lead to the generation of more random spatiotemporal patterns of microstates.

## Figures and Tables

**Table 1:** Demographic characterization and database description.

Subject	Population	Sex (Male/Female)	Age	MMSE	Duration of Disease (Months)
HC	29	11/18	67.9 (5.4)	30	--
FTD	23	14/9	63.6 (8.2)	22.17 (8.22)	23 (9.35)
AD	36	13/23	66.4 (7.9)	17.75 (4.5)	25 (9.88)

**Table 2:** Spatial correlation between microstate template maps across groups.

Groups	Map-A	Map-B	Map-C	Map-D	Average Pre-Comparison
HC vs. FTD	0.922	0.938	0.913	0.887	0.915
HC vs. AD	0.914	0.947	0.944	0.924	0.932
FTD vs. AD	0.993	0.985	0.983	0.952	0.978
Average per map	0.943	0.957	0.947	0.921	

**Table 3:** The detailed results of MS-NRI and MS-LZC for the HC, FTD, and AD groups across different IMFs.

Features	HC	FTD	AD	*p*1	*p*2	*p*3
EEG MSNRI	6.976 ± 1.623	5.809 ± 1.121	5.485 ± 1.614	0.008	0.152	<0.001
IMF2 MSNRI	6.545 ± 1.684	5.572 ± 1.109	5.288 ± 1.441	0.039	0.216	0.0014
IMF3 MSNRI	6.726 ± 1.559	5.596 ± 1.138	5.273 ± 1.531	0.009	0.152	<0.001
IMF4 MSNRI	7.103 ± 1.648	5.952 ± 1.329	5.689 ± 1.774	0.012	0.283	0.001
IMF5 MSNRI	7.606 ± 1.717	6.310 ± 1.447	6.027 ± 1.833	0.009	0.338	0.001
EEG MSLZC	0.394 ± 0.011	0.403 ± 0.008	0.405 ± 0.010	0.016	0.267	0.0014
IMF2 MSLZC	0.396 ± 0.013	0.405 ± 0.009	0.407 ± 0.009	0.041	0.2871	0.0016
IMF3 MSLZC	0.397 ± 0.011	0.404 ± 0.007	0.407 ± 0.010	0.034	0.115	0.0016
IMF4 MSLZC	0.390 ± 0.016	0.401 ± 0.012	0.404 ± 0.012	0.025	0.309	0.0022
IMF5 MSLZC	0.391 ± 0.014	0.397 ± 0.015	0.402 ± 0.015	0.136	0.247	0.0099

*p*1: Statistical significance between HC and FTD, *p*2: Statistical significance between FTD and AD, *p*3: Statistical significance between HC and AD.

**Table 4:** Evaluation metrics for the multivariate linear regression model predicting MMSE scores using resting-state EEG indices for all participants.

Methods	Participants	Features	Model	R^2^	MSE	RMSE	MAE
Microstate dynamics	HC, FTD, AD	MSNRI, MSLZC, Duration, Occurrence, Coverage	Multiple linear regression	0.388	22.888	4.784	3.511
CEEMD-enhanced microstate dynamics	HC, FTD, AD	CEEMD-enhanced (MSNRI, MSLZC, Duration, Occurrence, Coverage)	Multiple linear regression	0.702	11.158	3.340	2.555

**Table 5:** Evaluation metrics for the multivariate linear regression model predicting MMSE scores based on resting-state EEG indices for the cognitive impairment group.

Methods	Participants	Features	Model	R^2^	MSE	RMSE	MAE
Microstate dynamics	FTD, AD	MSNRI, MSLZC, Duration, Occurrence, Coverage	Multiple linear regression	0.297	13.543	3.680	2.877
CEEMD-enhanced microstate dynamics	FTD, AD	CEEMD-enhanced (MSNRI, MSLZC, Duration, Occurrence, Coverage)	Multiple linear regression	0.940	1.160	1.077	0.807

**Table 6:** Comparison with previous studies on the EEG-based prediction of MMSE scores.

Authors	Subjects	Methods	Model	Validation	R^2^	*r*	RMSE
Wei et al. (2024) [37]	160 (MCI, dementia)	Time/spectral-domain features	LASSO	LOSO CV	0.230	--	2.680
Jiao et al. (2023) [20]	330 (AD)	Time/spectral/microstate features	Random forest regression	10-fold CV	0.820	--	--
Doan et al. (2021) [38]	122 (HC, dementia)	Spectral-domain features	Linear regression	5-fold CV	--	0.680	--
Jesus et al. (2021) [39]	89 (AD)	Spectral/coherence	Random forest regression	5-fold CV	--	0.348	1.682
Zorick et al. (2020) [40]	20 (elderly)	Multifractal detrended fluctuation analysis	Classification and Regression Trees	LOSO CV	--	0.650	--
Si et al. (2023) [41]	88 (HC, FTD, AD)	Functional connection	Multiple linear regression	LOSO CV	--	0.274	--
Our study	88 (HC, FTD, AD)	CEEMD- enhanced microstate dynamics	Multiple linear regression	LOSO CV	0.702	--	3.340
	59 (FTD, AD)				0.940	--	1.077

*r*: Pearson correlation coefficients. LASSO: the least absolute shrinkage selection operator. CV: cross-validation. RMSE: the root mean squared error.

## Data Availability

These data were derived from the following resources available in the public domain: https://openneuro.org/datasets/ds004504, accessed on 17 October 2023 [2].

## Supplementary Materials

The following supporting information can be downloaded at: https://www.mdpi.com/article/10.3390/brainsci14050487/s1, Table S1: Detailed MMSE scores for each subject.
