# Supplementary material for: Beyond Frequency Bands: Complementary-Ensemble-Empirical-Mode-Decomposition-Enhanced Microstate Sequence Non-Randomness Analysis for Aiding Diagnosis and Cognitive Prediction of Dementia
Source: Brain Sci. 2024 May 11;14(5):487. doi: 10.3390/brainsci14050487 (PMC11118442; doi:10.3390/brainsci14050487)
Supplement: Supplementary file 1 [file brainsci-14-00487-s001.zip › brainsci-2970052-supplementary.pdf]

**Table S1.** Detailed MMSE scores for each subject

| Subject | Group | MMSE | Subject | Group | MMSE | Subject | Group | MMSE |
|---|---|---|---|---|---|---|---|---|
| 1 | HC | 30 | 31 | FTD | 24 | 61 | AD | 23 |
| 2 | HC | 30 | 32 | FTD | 25 | 62 | AD | 20 |
| 3 | HC | 30 | 33 | FTD | 22 | 63 | AD | 22 |
| 4 | HC | 30 | 34 | FTD | 22 | 64 | AD | 18 |
| 5 | HC | 30 | 35 | FTD | 20 | 65 | AD | 20 |
| 6 | HC | 30 | 36 | FTD | 18 | 66 | AD | 14 |
| 7 | HC | 30 | 37 | FTD | 22 | 67 | AD | 18 |
| 8 | HC | 30 | 38 | FTD | 20 | 68 | AD | 14 |
| 9 | HC | 30 | 39 | FTD | 22 | 69 | AD | 6 |
| 10 | HC | 30 | 40 | FTD | 24 | 70 | AD | 23 |
| 11 | HC | 30 | 41 | FTD | 22 | 71 | AD | 14 |
| 12 | HC | 30 | 42 | FTD | 22 | 72 | AD | 4 |
| 13 | HC | 30 | 43 | FTD | 18 | 73 | AD | 22 |
| 14 | HC | 30 | 44 | FTD | 20 | 74 | AD | 20 |
| 15 | HC | 30 | 45 | FTD | 18 | 75 | AD | 16 |
| 16 | HC | 30 | 46 | FTD | 27 | 76 | AD | 20 |
| 17 | HC | 30 | 47 | FTD | 20 | 77 | AD | 20 |
| 18 | HC | 30 | 48 | FTD | 24 | 78 | AD | 18 |
| 19 | HC | 30 | 49 | FTD | 26 | 79 | AD | 16 |
| 20 | HC | 30 | 50 | FTD | 26 | 80 | AD | 20 |
| 21 | HC | 30 | 51 | FTD | 24 | 81 | AD | 16 |
| 22 | HC | 30 | 52 | FTD | 24 | 82 | AD | 20 |
| 23 | HC | 30 | 53 | AD | 16 | 83 | AD | 22 |
| 24 | HC | 30 | 54 | AD | 22 | 84 | AD | 20 |
| 25 | HC | 30 | 55 | AD | 14 | 85 | AD | 20 |
| 26 | HC | 30 | 56 | AD | 20 | 86 | AD | 18 |
| 27 | HC | 30 | 57 | AD | 22 | 87 | AD | 22 |
| 28 | HC | 30 | 58 | AD | 14 | 88 | AD | 9 |
| 29 | HC | 30 | 59 | AD | 20 | | | |
| 30 | FTD | 20 | 60 | AD | 16 | | | |
